# Controllable photoluminescence enhancement of CdTe/CdS quantum dots thin films incorporation with Au nanoparticles

**DOI:** 10.1186/s11671-015-0833-3

**Published:** 2015-03-12

**Authors:** Hongyu Wang, Ling Xu, Renqi Zhang, Zhaoyun Ge, Wenping Zhang, Jun Xu, Zhongyuan Ma, Kunji Chen

**Affiliations:** National Laboratory of Solid State Microstructure and School of Electronic Science and Engineering, Nanjing University, Nanjing, 210093 People’s Republic of China; Nanjing University of Posts and Telecommunications, Nanjing, 210003 Jiangsu Province People’s Republic of China

**Keywords:** Photoluminescence, Finite-difference time-domain, Plasmon, Quantum dots

## Abstract

Au nanoparticles (Au NPs)/CdTe/CdS QDs nanocomposite films were fabricated by deposition of Au NPs and layer-by-layer self-assembly of colloidal CdTe/CdS QDs. Photoluminescence (PL) spectra showed that Au NPs incorporation resulted in an increase of PL intensity about 16-fold compared with that of the samples without Au NPs. PL enhancement of Au NPs/CdTe/CdS QDs nanocomposite films can be controlled by tuning the thickness of spacer layer between the metal nanoparticles (MNPs) and QDs. Optical absorption spectra exhibited the incorporation of Au NPs boosted the absorption of Au NPs/CdTe/CdS QDs nanocomposite films. The results of finite-difference time-domain (FDTD) simulation indicated that the increased sizes of Au NPs resulted in stronger localization of electric field, which boosted the PL intensity of QDs in the vicinity of Au NPs. We thought that these were mainly attributed to localized SP enhancement effects of the Au NPs. Our experiment results demonstrated that Au NPs/QDs nanocomposite films would be a promising candidate for optoelectronic devices application.

**PACS**

78.55.-m; 82.33.Ln; 68.65.Hb

## Background

Semiconductor quantum dots (QDs) have been attracted much attention on the application of solid-state lighting and photovoltaics due to the unique optical properties. The use of QDs in such applications needs the investigation of obtaining highly efficient QDs. Recently, an effective approach by using the interaction of surface plasmon (SP) with QDs has been raised to enhance the photoluminescence (PL) emission of QDs [1,2]. There are two possible processes to enhance the PL emission: an increased emission rate and an increased excitation rate of the QDs, which are depended on the intensity of local electric field near metal nanoparticles (MNPs). The overall effect of SP at work is determined by the competition between emission enhancement, excitation enhancement, and quenching [3]. Some research groups have demonstrated the application of MNPs in optoelectronic devices based on the SP resonance effects [4-7]. The effect of the size of MNPs and the thickness of spacer layer between MNPs and QDs on the plasmonic interaction has rarely been demonstrated.

In this paper, the Au NPs/QDs nanocomposite films were fabricated using a layer-by-layer self-assembly technique. The PL spectra and fluorescence decay of QDs film were measured in the absence and presence of Au NPs. The influence of the spacer layer thickness on the variations of PL intensity of QDs was analyzed. In addition, the effect of the size of MNPs on the plasmonic interaction was studied. The local electric field in the vicinity of Au NPs with different sizes was modeled by our finite-difference time-domain (FDTD).

## Methods

Au NPs with different sizes were formed on quartz substrates by ion sputtering in a vacuum chamber and then high temperature rapid thermal annealing (RTA) process. 3-Mercaptopropionic acid (MPA) capped CdTe/CdS QDs were synthesized via a modified reported method [8]. CdTe/CdS QDs were assembled on Au NPs/quartz substrate by layer-by-layer method [9]. Positively charged poly(diallyldimethylammonium chloride) (PDDA) and negatively charged poly(sodium 4-styrenesulfonate) (PSS) bilayer dielectric were served as spacer layer between the Au NPs and QDs. The thickness of spacer layer can be tuned and controlled by the number of PDDA/PSS bilayers.

The absorption spectra were recorded on 3100 UV-visible spectrometer. Room temperature PL spectra were measured by Jobin Yvon Fluolrolog-3 system equipped with a 450 W Xe lamp as the excitation sources. The fluorescence decay curves from time-resolved PL measurements were recorded by an Edinburgh FLS920 fluorescence spectrophotometer based on the time-correlated single photon counting (TCSPC). Topographic images were obtained by tapping mode AFM (Bruker, Nanoscope III-D) under a clean, dry ambient atmosphere.

## Results and discussion

**Figure 1:**
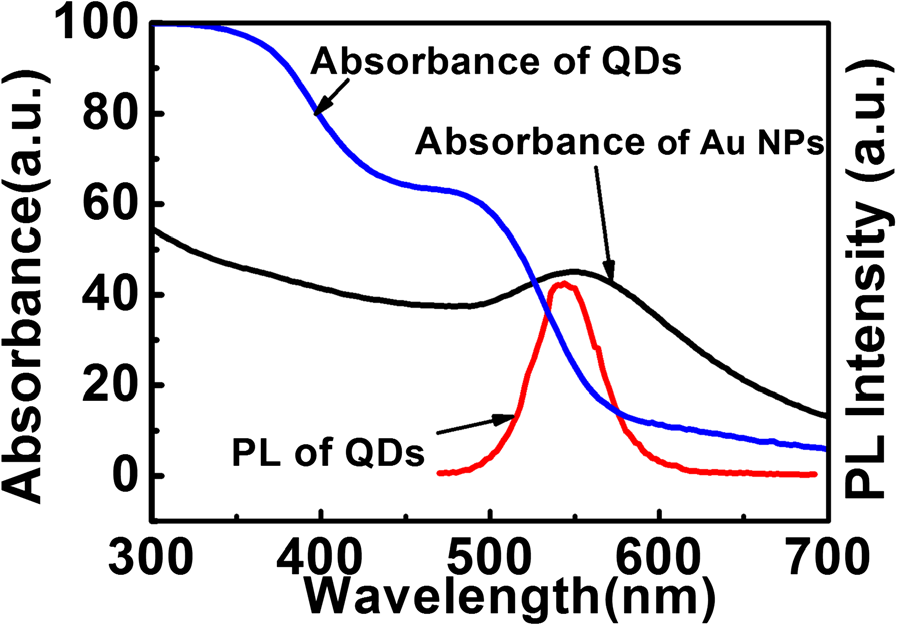
**Optical properties of Au NPs and QDs.** The absorption spectra of the Au NPs (black line) and QDs (blue line) together with the PL spectrum of the QDs (red line).

The absorption spectra of the Au NPs (absorption maximum at 550 nm) and QDs together with the PL spectrum of the QDs (544 nm) were shown in Figure 1. As we can see, the SP absorption band of Au NPs overlaps with the absorption and emission spectra of QDs, which might lead to an enhanced local electric field.

**Figure 2:**
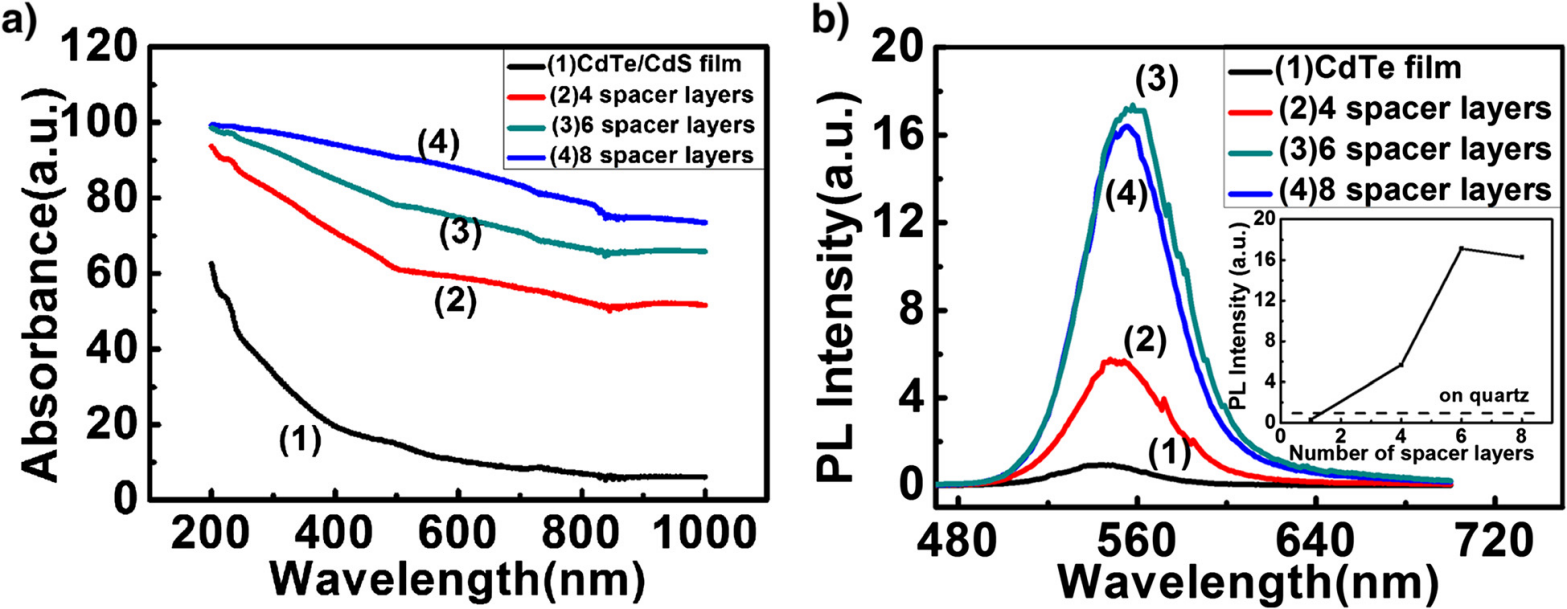
**UV–vis absorption spectra and PL spectra of Au NPs/QDs nanocomposite films with different number of PDDA/PSS bilayers. (a)** The absorbance spectra of samples. **(b)** The PL spectra of samples. The inset showed QDs film’ PL intensities versus the number of spacer layers between Au NPs and QDs.

In order to obtain controllable PL enhancement of Au NPs/CdTe/CdS QDs nanocomposite films, we prepared the dielectric films comprised of four, six, and eight PDDA/PSS bilayers between CdTe/CdS QDs film and Au NPs. The thickness of a single PDDA/PSS bilayer was approximately 1.4 nm according to our ellipsometry measurement and 8.4 nm for that of six PDDA/PSS bilayers. Figure 2a showed the UV–vis absorption spectra of the film with different spacer layer thicknesses. The absorbance of Au NPs/CdTe/CdS QDs films increased compared with that of QDs thin films without Au NPs. The maximum absorbance was obtained when the spacer layer was six PDDA/PSS bilayers. These results indicated that Au NPs incorporation boosted the absorption of QDs. The corresponding PL spectra of samples were presented in Figure 2b. The peak position of QDs film was almost no shift. Compared with the QDs without Au NPs, the PL intensity of QDs film with Au NPs increased dramatically. When the spacer layer was six PDDA/PSS bilayers, the PL intensity of CdTe/CdS QDs film showed 16-fold increase compared with that of QDs film without Au NPs. Further increase in the spacer thickness led to the decrease of PL intensity of QDs. For example, for eight bilayers, the enhancement of PL intensity of QDs dropped to a factor of 10. These results can be attributed to the competition between the energy transfer quenching and local electromagnetic field induced PL enhancement. At short spacer distance, the excited state electrons and holes of QDs can tunnel to Au NPs through nonradiative relaxation thereby quenching the fluorescence of QDs. As the distance increased, the nonradiative decay rate rapidly decreased [10]. In addition, the local electromagnetic field intensity was responsible for the PL enhancement of QDs. The electric field surrounding the Au NPs decayed with the separation distance increasing [10]. Thus, controllable PL enhancement of Au NPs/CdTe/CdS QDs film was achieved through tuning the spacer layer thicknesses between Au NPs and QDs. In this paper, the optimal spacer layer was six PDDA/PSS bilayers and at which the PL enhancement reached a maximum.

**Figure 3:**
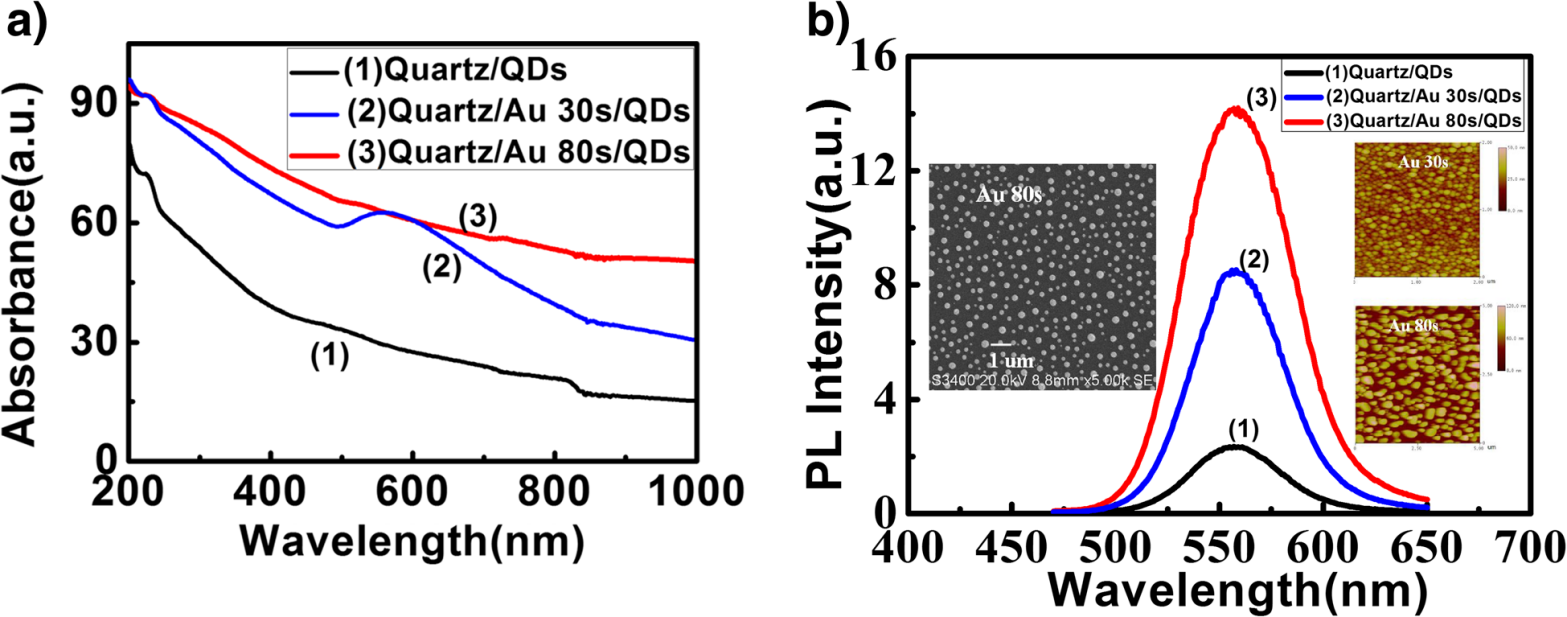
**UV–vis absorption and PL spectra of the Au NPs/QDs nanocomposite films with Au NPs in 30- and 80-s deposition times. (a)** The absorption spectra of Au NPs/QDs films with Au NPs in 30-s (blue line) and 80-s (red line) deposition times. **(b)** The PL spectra of Au NPs/QDs films with Au NPs in 30-s (blue line) and 80-s (red line) deposition times. The inset showed the AFM image of Au NPs in 30-s deposition times, AFM image of Au NPs in 80-s deposition times, and SEM image of Au NPs in 80-s deposition times.

The size of Au NPs was another important factor that controlled the plasmonic interaction and therefore the PL emission. In order to interpret this, Au was sputtered on the substrate in deposition times of 30 and 80 s. After a rapid high temperature annealing process, Au NPs with different sizes were obtained. The root mean square of roughness (RRMS) of Au NPs film and the sizes of Au NPs were determined from the surface morphological images of the atomic force microscopy (AFM), as shown in Table 1. Figure 3 showed that the intensity of absorbance and emission for the sample with Au NPs in 80-s deposition was higher compared with that of sample with Au NPs in 30-s deposition. These might attribute to the enhanced local electric field surrounding the Au NPs. The enhancement probably came from the overlap between the plasmonic region of Au NPs and the emission band, absorption band of QDs (Figure 1) [3,11,12].

**Table 1:** **The** ***R***_**RMS**_ **and the average size of Au NPs in different deposition times**

**Deposition times**	***R***_**RMS**_	**Average size**
30 s	4.85	100 ± 5 nm
80 s	21.9	300 ± 5 nm

**Figure 4:**
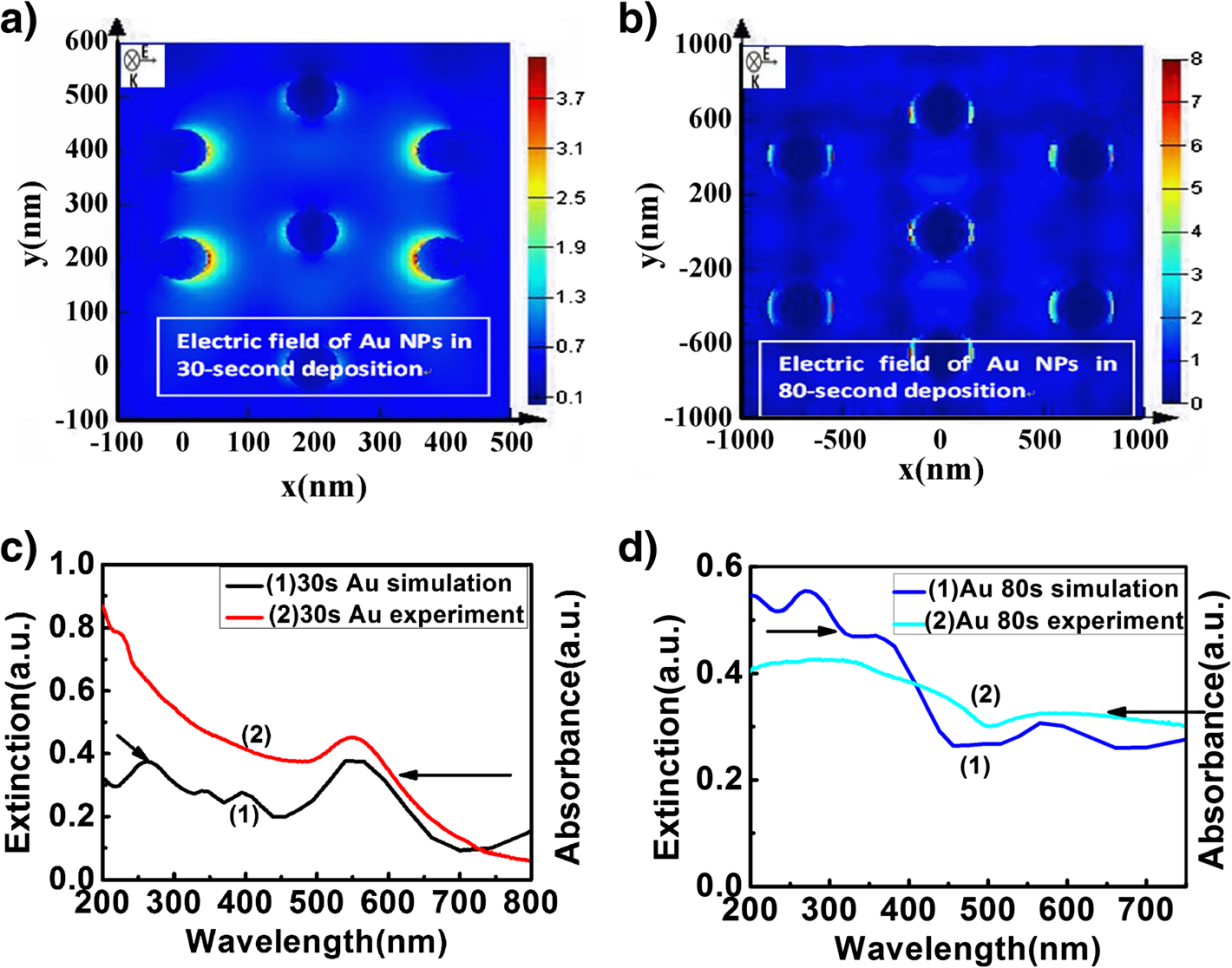
**The simulated electric field.** Simulated electric field surrounding the Au NPs **(a,b)** and simulated extinction spectra of Au NPs in 30- and 80-s deposition times **(c,d)**.

The enhanced local electric field surrounding the Au NPs was confirmed by our FDTD simulation. In our simulation structure, we considered z as the incident light direction, x was the polarization direction. We set the initial parameters, including the mesh type (uniform and mesh conformal variant 1), the smallest size (2 nm), the domain boundaries (PML), the simulation time (100 fs), the actual runtime (12 h), and the light source (plane wave from 200 to 800 nm). Figure 4 exhibited the simulated electric field surrounding the Au NPs and simulated extinction spectra of Au NPs in 30- and 80-s deposition times. From Figure 4c and d, we can see that the simulated extinction spectrum of Au NPs was consistent with the experimental results. In addition, the electric field surrounding the Au NPs in 30-s deposition was enhanced as high as 3.7-fold compared with the incident light (Figure 4a), however, that in 80-s deposition was enhanced eightfold (Figure 4b). These results indicated that the increased size of Au NPs resulted in stronger localization of electric field, which was induced by SP resonance [13]. Both theoretical and experimental results indicated that a strong local electric field in the vicinity of QDs was obtained when incorporating Au NPs. Therefore, the PL intensity of QDs film increased.

In order to verify the plasmonic resonances of Au NPs on QDs films, fluorescence lifetimes of samples were measured using a 375 nm excitation source at room temperature. The measurement data were fitted by a double exponential function. The equation can be expressed by *I*(*t*) = *B*_1_ exp(−*t*/*τ*_1_) + *B*_2_ exp(−*t*/*τ*_2_), where τ1, τ2, B1, and B2 are the decay times and their corresponding amplitudes. Average decay lifetime is given by equation:

$$ \overline{\tau}=\frac{B_1{\tau}_1^2+{B}_2{\tau}_2^2}{B_1{\tau}_1+{B}_2{\tau}_2} $$

**Figure 5:**
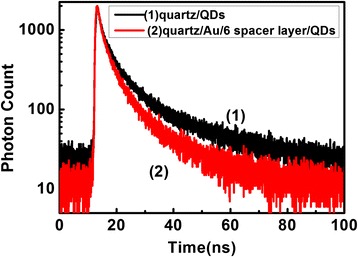
**Time-resolved photoluminescence decays of CdTe/CdS QDs film on the quartz substrate.** CdTe/CdS QDs film in the absence (black line) and presence (red line) of Au NPs.

Figure 5 showed that the PL lifetime at the emission peak of CdTe/CdS QDs was 9.1 ns. While in the presence of Au NPs, the lifetime decreased to 6.8 ns. The decreased lifetime may indicate the energy transfer between Au NPs and QDs due to the SP resonance. The results were consistent with the previous reports on plasmon coupled with CdTe/CdS QDs [14]. These might imply the energy transfer between the Au NPs and QDs by SP coupling. The efficiency of energy transfer was estimated approximately using the measured lifetimes. It was expressed as E = 1-τ’/τ, where τ’ and τ were the PL lifetimes of QDs in the presence and absence of Au NPs, respectively, according to references [15] and [16]. The efficiency of energy transfer between QDs and Au NPs was estimated to be ca. 26%.

**Figure 6:**
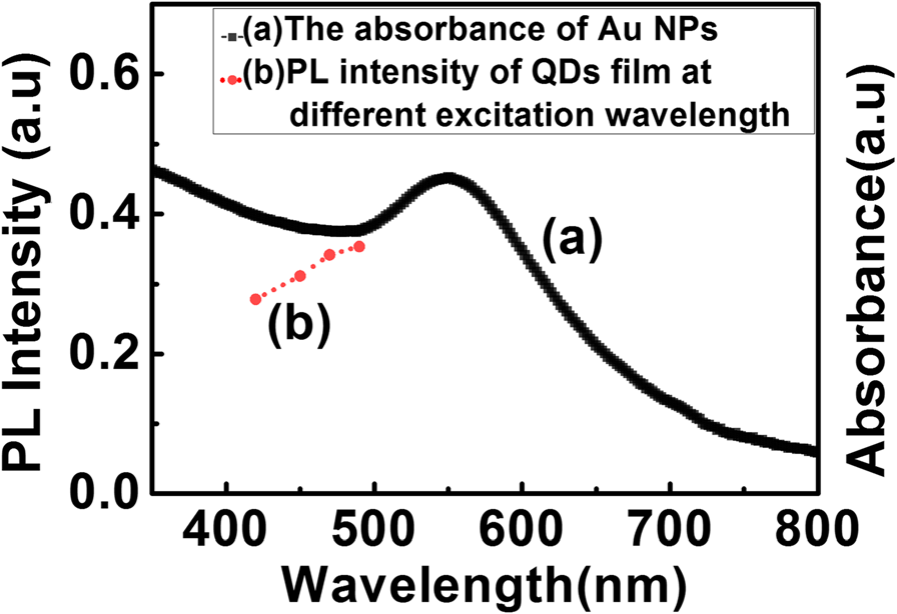
**PL intensity of QDs film at different excitation wavelength (red dot line) and the absorption spectrum of Au NPs (black line).**

To understand the origin of PL enhancement of QDs film, we analyzed the absorption spectra of the Au NPs and the optical properties of the QDs. The absorption band of Au NPs overlapped with that of QDs (Figure 1) leading to an effective energy transfer between the QDs and the metal surface plasmon field [17]. In the absence of Au NPs, the incident light was absorbed by the QDs. However, in the presence of Au NPs, the light excited the SP modes in the Au NPs, which enhanced the local electric field [3]. The enhanced absorption (Figure 2a) and the enhanced local electric field at the SP resonance wavelength induced the increasing of the excitation rate of QDs film, which led to the enhancement of PL emission intensity of QDs film. The plasmon enhanced PL at different excitation wavelength (420 to 490 nm) for the Au NPs/QDs nanocomposite films was examined (curve b as shown in Figure 6). When the excitation wavelength was close to the absorption peak of Au NPs (around 540 to 550 nm), the PL intensity of QDs film in the vicinity of Au NPs increased. A stronger coupling of the plasmon resonance to the excitation wavelength resulted in a larger enhancement effect in PL. Wiederrecht GP et al. also reported similar results that PL enhancement of CdSe QDs on Ag NPs film [3,18].

In addition, the absorption band of Au NPs overlapped with the emission band of QDs (Figure 1) inducing the enhanced local electric field, which might result in the increase of the emission rate of QDs. Similar results were also reported from another study conducted by Yang Y [12]. In brief, Au NPs incorporation resulted in an increase of optical properties of QDs film compared with that of the samples without Au NPs.

**Figure 7:**
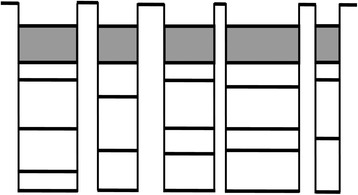
**The sketch of electron levels in QDs film.**

Many groups have investigated the energy levels for composite thin films or the hybrids NPs. Wei Wu et al. have reported the synthesis of the Au-SnO2 hybrid NPs and analyzed the band configuration of Au-SnO2 composite NPs [19]. Here in our case, CdTe/CdS QDs were assembled on Au NPs/quartz substrate by layer-by-layer method. CdTe/CdS QDs and Au NPs were separated by different number of spacer layers. Therefore, we thought the energy level of CdTe/CdS QDs doesn’t affect that of Au NPs. As for CdTe/CdS QDs, the evolution of the electron state of a nanocrystal from an individual to a collective state may occur when the numbers of layers decrease. That is when a close-packed quantum dot ensemble was formed the evolution from a discrete electron state to collective minisubbands of energy level may occur. This effect is similar with the formation of mimibands in superlattice of quantum wells [20]. The sketch of electron levels in QDs films were shown in the Figure 7.

## Conclusions

In conclusion, we fabricated Au NPs/CdTe/CdS QDs nanocomposite films by deposition of Au NPs and layer-by-layer self-assembly of colloidal CdTe/CdS QDs. PL spectra showed the incorporation of Au NPs induced an increase of PL intensity about 16-fold compared with that of the samples without Au NPs due to the enhanced local electric field surrounding the Au NPs. Controllable PL enhancement of Au NPs/CdTe/CdS QDs film was obtained through tuning the spacer layer thicknesses between Au NPs and QDs, which was attributed to the competition between the energy transfer quenching and local electromagnetic field. The results of finite-difference time-domain (FDTD) simulation indicated that the increased sizes of Au NPs resulted in stronger localization of electric field, which boosted the PL intensity of QDs film in the vicinity of Au NPs. In summary, PL intensity of QDs film was enhanced due to plasmonic interaction. Therefore, higher efficiency metal/QDs nanocomposite films may have potential application in optoelectronic device.

## Abbreviations

Au NPs/CdTe/CdS QDs: Au nanoparticles/CdTe/CdS quantum dots
FDTD: finite-difference time-domain
PL: photoluminescence
MNPs: metal nanoparticles
SP: surface plasmon
